# Non-availability of anesthesia scavenging system and decontamination of the outflow gas from the anesthesia machine during this COVID-19 pandemic

**DOI:** 10.1186/s42077-020-00096-5

**Published:** 2020-10-06

**Authors:** Arimanickam Ganesamoorthi, Vinodhadevi Vijayakumar, Vasanthakumar Vellaichamy, Gopalakrishnan Panneerselvam

**Affiliations:** Department of Anesthesiology and Critical Care, Meenakshi Hospital, Thanjavur, 613005 India

**Keywords:** Anesthesia scavenging system, Active gas scavenging system, Heat moisture exchanger bacterial-viral filter (HMEF), High-efficiency particulate air (HEPA) filter, Coronavirus disease (COVID-19) pandemic

To the Editor,

It is recommended that breathing system filters should be incorporated in the expiratory limb of any ventilator, when used on a patient with severe acute respiratory syndrome (SARS) (Wilkes, 2011; Mechanical ventilation of SARS patients, 2003). The breathing circuit filters having bacterial and viral filtration efficiencies of 99.97% or greater will offer protection equal to or better than high-efficiency particulate air (HEPA) filters (Wilkes, 2011; Mechanical ventilation of SARS patients, 2003). These filtering barriers are placed at three locations to reduce the contagion during anesthesia using a circle system with CO_2_ absorber:

1. Between the tracheal tube and the breathing circuit (Wilkes, 2011; Mechanical ventilation of SARS patients, 2003; Infection prevention and control guidelines for anesthesia care, 2020)
2. Between the inspiratory limb of the circle system and the CO_2_ absorber (Infection prevention and control guidelines for anesthesia care, 2020)
3. Between the expiratory limb of the circle system and the CO_2_ absorber (Wilkes, 2011; Mechanical ventilation of SARS patients, 2003; Infection prevention and control guidelines for anesthesia care, 2020)

Apart from the above precautions, usage of anesthesia scavenging system is recommended while anesthetizing a suspect/confirmed COVID-19 patient to prevent the potential contamination of the operating room with SARS-CoV-2 virus (Malhotra et al., 2020). In many of the low- and middle-income countries (LMIC), it is not uncommon to work with anesthesia workstations without an anesthesia scavenging system. One of the methods suggested is that a corrugated tubing can be applied to the scavenging port and can be dipped in a bucket with 1% hypochlorite solution (Malhotra et al., 2020). While using such technique, suitable personal protective equipment should be used while handling the hypochlorite solution and direct contact with the skin and eyes should be avoided (Malhotra et al., 2020).

In our hospital, we are using the Drager Fabius Plus anesthesia workstation without an anesthesia scavenging system/active gas scavenging (AGS). We attached a HEPA filter or a HMEF (heat moisture exchanger bacterial/viral filter) to the AGS (active scavenging system) port of the anesthesia machine (Fig. 1). HMEF/HEPA filter at the AGS would filter 99.97% of virus particles before the exhaled gas enters the operating room atmosphere. We also observed that placing the HMEF/HEPA filter at the AGS had not altered the measured airway pressures like positive end-expiratory pressure (PEEP) or peak airway pressure during ventilation. We replace the HMEF/HEPA filter connected to the AGS with a new one every 24 h. If the PEEP value increases without any patient factors, a change of the HMEF/HEPA filter should also be considered. The filter will be removed, and the PEEP behavior will be observed for 3 breaths; if the measured value normalizes, then the filter has to be changed (Wilkes, 2011; COVID-19: Usage of Dräger anaesthesia devices for long-term ventilation dated 19 may 2020, 2020). The HMEF/HEPA filter can be connected to the AGS, even if the anesthesia machine ventilator is used for prolonged ventilation of critically ill patients, like an ICU ventilator when a shortage arises in this COVID-19 pandemic (COVID-19: Usage of Dräger anaesthesia devices for long-term ventilation dated 19 may 2020, 2020). The HMEF/HEPA filter does not serve as an anesthesia gas scavenging system, but definitely filters bacteria and virus, which is the prime concern during this COVID-19 pandemic.

**Fig. 1:**
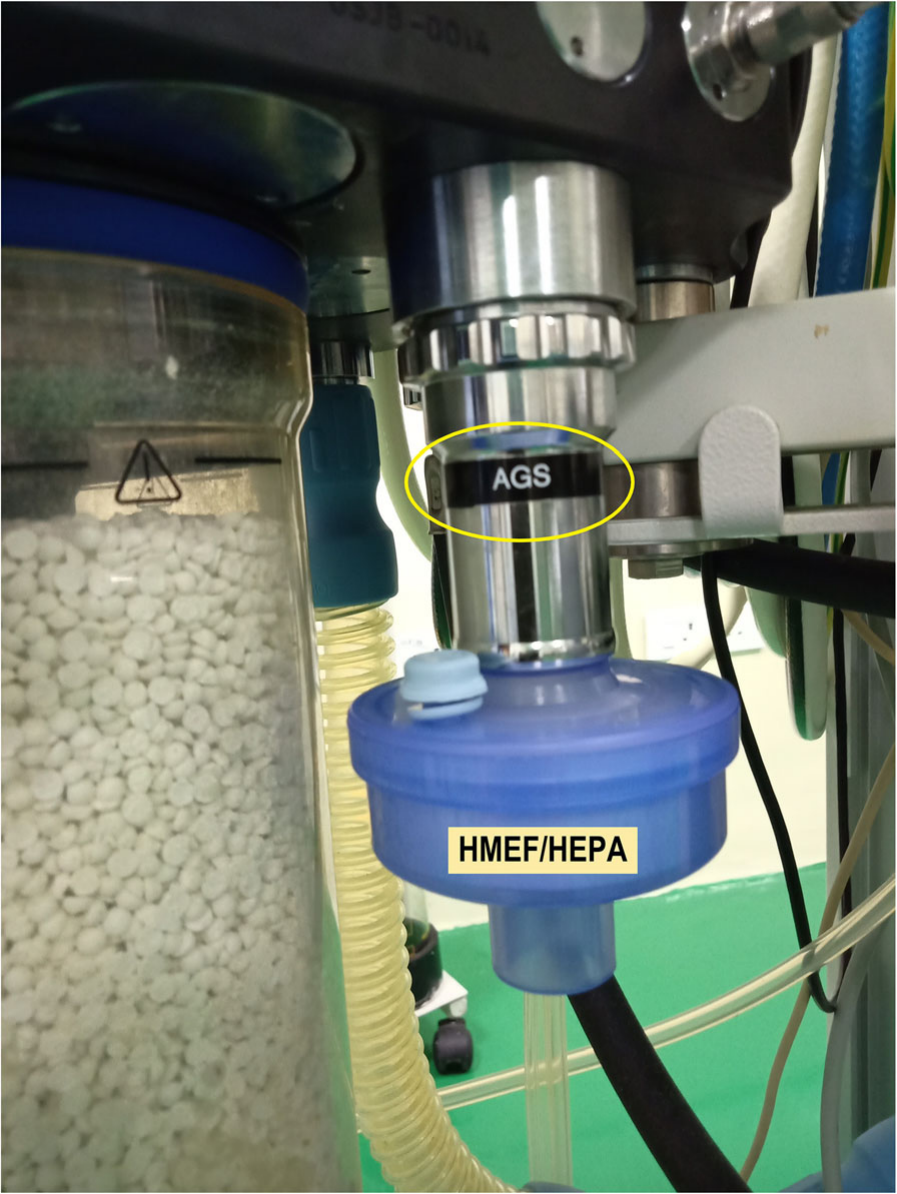
Drager Fabius Plus anesthesia workstation—the heat moisture exchanger bacterial-viral filter (HMEF) is attached to the active gas scavenging (AGS) port (labeled on the machine by the manufacturer). HEPA, high-efficiency particulate air filter

To conclude, in the current COVID-19 pandemic in operating rooms where anesthesia scavenging system is unavailable, placing an HMEF/HEPA filter at the AGS port is an easy and simple method which adds to the safety and effectively reduces the contamination of the operating room with the SARS-CoV-2 virus.

## Data Availability

Not applicable

## Abbreviations

SARS: Severe acute respiratory syndrome
HEPA filter: High-efficiency particulate air filter
CO_2_: Carbon dioxide
COVID-9: Coronavirus disease 2019
SARS-CoV-2: Severe acute respiratory syndrome coronavirus 2
LMIC: Low- and middle-income countries
AGS: Active gas scavenging
HMEF: Heat moisture exchanger bacterial/viral filter
PEEP: Positive end-expiratory pressure
